# Adaptive Smoothness Constraint Ionospheric Tomography Algorithm

**DOI:** 10.3390/s20082404

**Published:** 2020-04-23

**Authors:** Debao Wen, Dengkui Mei, Yanan Du

**Affiliations:** 1 School of Geographical Sciences, Guangzhou University, Guangzhou 510006, China; yndu@gzhu.edu.cn; 2 School of Traffic and Transportation Engineering, Changsha University of Science & Technology, Changsha 410004, China; mei_dk@163.com

**Keywords:** adaptive smoothness constraint, ionospheric tomography, ill-posed problem, ionospheric electron density, slant total electron content

## Abstract

Ionospheric tomography reconstruction based on global navigation satellite system observations is usually an ill-posed problem. To resolve it, an adaptive smoothness constraint ionospheric tomography algorithm is proposed in this work. The new algorithm performs an adaptive adjustment for the constrained weight coefficients of the tomography system. The computational efficiency and the reconstructed quality of ionospheric imaging are improved by using the new algorithm. A numerical simulation experiment was conducted in order to validate the feasibility and superiority of the algorithm. The statistical results of the reconstructed errors and the comparisons of ionospheric profiles confirmed the superiority of the new algorithm. Finally, the new algorithm was successfully applied to reconstruct three-dimensional ionospheric images under geomagnetic quiet and geomagnetic disturbance conditions over Hunan province. The tomographic results are reasonable and consistent with the general behavior of the ionosphere. The positive and negative phase storm effects are found during geomagnetic storm occurrence.

## 1. Introduction

The ionosphere is the upper atmosphere between 60 km and 1000 km above the earth. The temporal and spatial variations of the ionosphere affect the propagation of radio signals [1,2,3], especially during the occurrence of geomagnetic storms. Therefore, it is necessary to detect and understand the characteristics of ionosphere activity under different geomagnetic conditions. Ionospheric electron density (IED) and vertical total electron content (VTEC) are the key physical parameters for the studies of the ionospheric activity [4]. However, VTEC models can only reconstruct the two-dimensional ionospheric structure in the cross-section of latitude and longitude. In late 1980s, Austen et al. [5] introduced the computerized ionospheric tomography technique to reconstruct the IED distributions by using the observations of the navy navigation satellite system. However, the longitudinal variation is ignored because the ground receivers are aligned along the fixed longitudinal chain. To reconstruct the ionospheric structure, three-dimensional ionospheric variations need to be studied. Using simulated high-orbit satellite data, Kunitsyn et al. [6] successfully reconstructed the three-dimensional ionospheric structure. The advent and development of the global navigation satellite system (GNSS) has provided a new avenue for monitoring and studying ionosphere. The construction of more ground-based GNSS networks and the application of more space-based GNSS observations make it possible to reconstruct the three-dimensional IED distributions. Therefore, the GNSS-based ionospheric tomography technique has attracted the interest of many scholars [7,8,9,10,11,12,13,14,15,16]. Theoretical and experimental research has been conducted, and some meaningful results have been achieved. Leitinger et al. [17] further validated the feasibility of the GNSS-based ionospheric tomography technique by using an actual GNSS experiment. Some scholars studied the ionospheric response to geomagnetic storms by using GNSS-based ionospheric tomography [18,19]. Meanwhile, some efficient algorithms have been proposed to improve the accuracy and resolution of the reconstructed ionospheric images [20,21].

Due to the nonuniform and sparsity of ground GNSS observation stations, the input data of the GNSS-based ionospheric tomography are usually insufficient. This fact makes it an ill-posed problem [22]. To resolve this issue, scholars have studied some tomographic algorithms in recent years [12,14,22,23,24,25,26,27,28,29]. On the whole, these algorithms are divided into the iterative method and the non-iterative method. In the iterative method, the multiplicative algebraic reconstruction technique (MART) is usually applied to reconstruct the IED distributions due to its positive results [25]. The MART solves the ill-posed problem by adding an initial value to each voxel. For the slow convergence speed of the MART, Zhao et al. [20] presented an adaptive MART (AMART) algorithm. By adaptively updating the relaxation factor, the algorithm improves the computational efficiency to some extent. However, for the two algorithms, the final results of those voxels without rays traversing them are the same as the initial values, which are obtained from empirical ionosphere models such as the international reference ionosphere (IRI) model. Therefore, the tomographic accuracy is limited [4]. To overcome the disadvantages of the MART, smoothness constraint MART (SCMART) algorithm has been proposed by considering the correlation between the variation of IED and the distance [30]. The algorithm improves the quality of the reconstructed IED images by imposing a smoothing constraint on the tomographic system. However, the horizontal constraint weight coefficients are invariable in the iterative process, and the altitudinal constraint is not considered. A round of iteration is considered when all the rays intersecting the voxels are involved in one computation. Considering the variation of IED after each round of iteration, an adaptive smoothness constrains MART (ASCMART) is presented in this work. The new algorithm adaptively adjusts the horizontal and altitudinal constraint weight coefficients according to the results of the last round of iteration. A numerical simulation scheme was devised to validate the feasibility and superiority of the new algorithm. Finally, the algorithm was applied to reconstruct the three-dimension distribution of the IED over Hunan province under geomagnetic quiet and geomagnetic disturbance conditions by using the GNSS observations of the continuously operating reference stations (CORS). The reconstructed error statistics and the comparisons of the hmF2 and NmF2 further verified the superiority of the new algorithm.

## 2. Tomographic Theory

The slant total electron content (STEC) along the ray path can be expressed as:

(1) $$STEC=\int_{p}N_{e}\left(s\right)ds$$

where $N_{e}\left(s\right)$ is the IED distribution, and p represents the ray path between a satellite and a receiver. 

**Figure 1:**
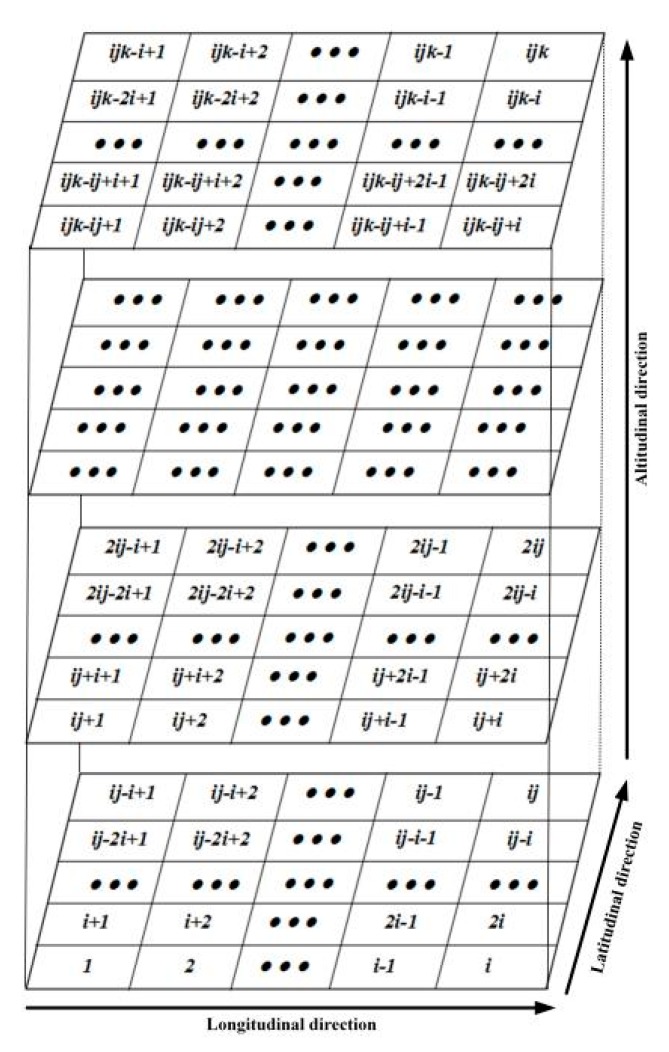
Sketch map of the partition and numbers of the voxel.

To reconstruct the three-dimensional IED distribution using the STEC data, the selected region is divided into small cubic voxels. The ionosphere is discretized into i layers, j layers, and k layers in longitudinal, latitudinal, and altitudinal directions, respectively. The number sequences of the voxels are shown in Figure 1. Then, Equation (1) can be simplified as:

(2) $$STEC_{c}=\overset{n}{\underset{d=1}{\sum }}A_{cd}x_{d}+e_{c}$$

Equation (2) is generally represented as a matrix notation:

(3) $$y_{m\times 1}=A_{m\times n}x_{n\times 1}+e_{m\times 1}$$

where m represents the number of the input STEC data; n is the total voxel numbers, which equals ijk; y is the vector of the m known STEC measurements; A is a coefficient matrix, the element $A_{cd}$ is the length of the cth path in the dth voxel; x is a vector of the unknown IED; and e is a column vector related to the discretization errors and measurement noises [31]. 

## 3. Adaptive Smoothness Constraint MART

To solve the ill-posed problem of ionospheric tomographic inversion, it is necessary to select a reasonable algorithm. Compared with the conventional MART algorithm, the SCMART is very attractive, since it overcomes the disadvantages of the MART to some extent. In general, the MART algorithm is iterated cyclically and can be implemented as follows [25]:

(4) $$x_{d}^{\left(p+1\right)}=x_{d}^{\left(p\right)}\cdot {\left(\frac{y_{c}}{\langle A_{c},x^{\left(p\right)}\rangle }\right)}^{\frac{\lambda A_{cd}}{\langle A_{c},A_{c}\rangle }}$$

where $x_{d}^{\left(p\right)}$ refers to the dth member of the vector x after the pth round iteration; $y_{c}$ is the cth row of the vector y; $A_{c}$ is the cth row of the matrix A; and λ is the relaxation parameter, which is set to be 0.2 in this work.

In general, the IED variation is smooth and continuous, and the correlation of IED between voxels increases with the distance shortening in the same horizontal layer. The Gauss weighted function is used to create horizontal constraints in the SCMART [30]. For each horizontal layer, we construct a constraint matrix, which is given as

(5) $$H_{L}=\left(\begin{array}{ccccc} -1 & h_{12} & h_{13} & \cdots & h_{1q}\\ h_{21} & -1 & h_{23} & \cdots & h_{2q}\\ \cdots & \cdots & \ddots & \cdots & \cdots \\ h_{q1} & h_{q2} & h_{q3} & \cdots & -1 \end{array}\right)$$

(6) q=i×j

where q is the number of voxels in one horizontal layer; and L refers to the $L_{th}$ layer in the altitudinal direction. In the SMART, the element of $H_{L}$ is calculated using the following equation:

(7) $$h_{cd}=\frac{e^{-\left(D_{cd}^{2}/2\sigma^{2}\right)}}{\sum_{c=1}^{i}\sum_{d=1}^{j}e^{-\left(D_{cd}^{2}/2\sigma^{2}\right)}}$$

where $D_{cd}$ is the distance between the cth voxel and the dth voxel; and σ is the smoothing operator. 

Since the distance $D_{cd}$ among voxels is stable, we can see that the $h_{cd}$ is invariant in each round of iteration. It influences the imaging quality of IED and the computational efficiency. Taking into account the change of IED after each round of iteration, the $h_{cd}$ can be adaptively adjusted according to the results of the last round of iteration in the ASMART, and then Equation (7) is changed as

(8) $$h_{cd}^{\left(p\right)}=\frac{e^{-\left({\left(Q_{cd}^{\left(p-1\right)}\right)}^{2}/2\sigma^{2}\right)}}{\sum_{c=1}^{i}\sum_{d=1}^{j}e^{-\left({\left(Q_{cd}^{\left(p-1\right)}\right)}^{2}/2\sigma^{2}\right)}}$$

In Equation (8), Q is the adaptive adjustment factor, which can be describe as

(9) $$Q_{cd}^{\left(p-1\right)}=D_{cd}\times \frac{x_{d}^{\left(p-1\right)}}{x_{c}^{\left(p-1\right)}}$$

Considering all the horizontal layers, the horizontal constraint can be represented as a matrix notation:

(10) Hx=0

According to Equation (5), the horizontal constraint matrix is written as

(11) $$H=\left(\begin{array}{ccccc} H_{1} & & & & \\ & H_{2} & & & \\ & & \ddots & & \\ & & & H_{k-1} & \\ & & & & H_{k} \end{array}\right)$$

In the altitudinal direction, the variation of IED is usually smooth and continuous, and the adaptive constraint matrix G can then be created using Equation (12). There are ij(k−1) lines and ijk columns in matrix G.

(12)
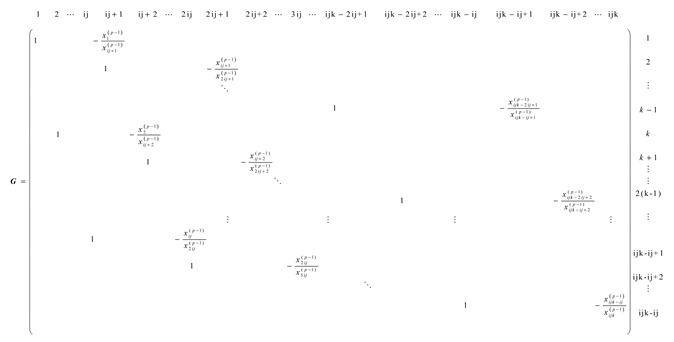


Therefore, the altitudinal constraint can be represented as a matrix notation:

(13) Gx=0

Combining Equation (3) with Equations (10) and (13), we can obtain

(14) $$\left(\begin{array}{c} A\\ H\\ G \end{array}\right)x+\left(\begin{array}{c} e\\ 0\\ 0 \end{array}\right)=0$$

Equation (14) is expressed as

(15) $$Y_{\left(m+2n-ij\right)\times n}x_{n\times 1}+E_{\left(m+2n-ij\right)\times 1}=0$$

## 4. Algorithm Validation

**Figure 2:**
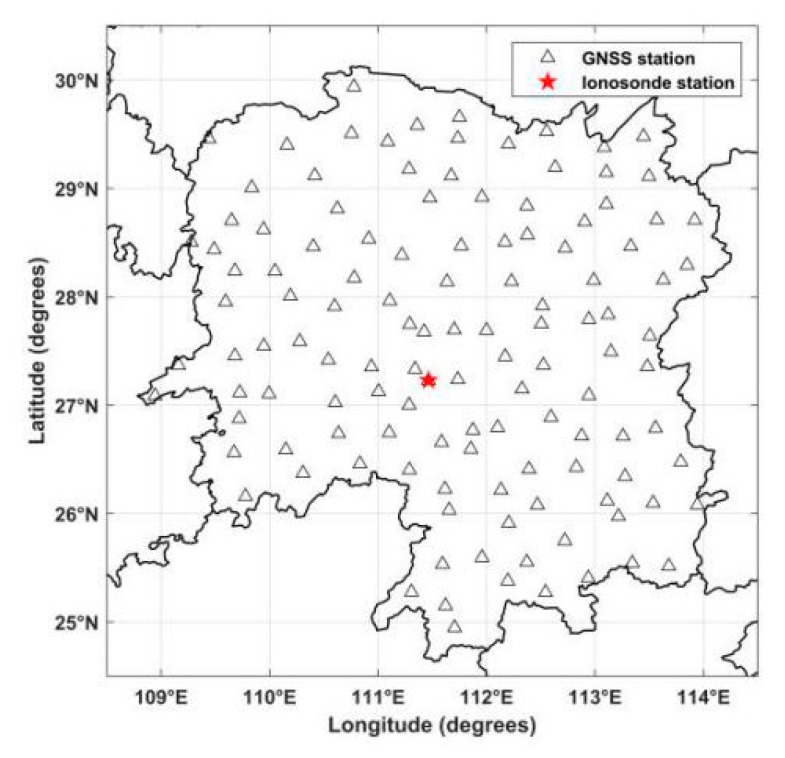
Distribution of the continuously operating reference network GNSS stations and ionosonde station in Hunan province.

The ASCMART is an improvement on the SCMART. However, the SCMART is an improvement of the MART. Instead of the AMART, the MART is introduced to compare with the ASCMART and the SCMART in this work. To confirm the feasibility and superiority of the new algorithm over the MART and the SCMART methods, a numerical simulation scheme was devised. The simulation scenario is as follows:

(1) The reconstructed region was Hunan province of southern China (24.3° N–30.3° N, 108.3° E–114.3° E), and the altitude ranged from 100 km to 1000 km. The discretized intervals are 0.5° in the longitudinal and latitudinal directions. Different from the equal spacing division in the horizontal direction, the unequal spacing division was adopted in the altitudinal direction. The discretized interval was 20 km in the altitudinal range from 200 km to 400 km. However, the discretized interval was 50 km in other altitudinal ranges. 

(2) In the numerical simulation experiment, the selected time period was from 00:00 UT to 00:30 UT on 20 June 2015. 

(3) The coordinates of the observed satellites and the 124 CORS stations in Hunan Province were used to calculate the crossing distance between the rays and the corresponding voxels, and the coefficient matrix was then constructed. The distributions of the 124 CORS GNSS stations and an ionosonde station were shown in Figure 2.

To mimic $Y_{simu}$, the true IED distributions were generated from IRI 2016 model. Therefore, the STEC values without noise can be computed using the following formula:

(16) $$Y_{simu}=AX_{simu}$$

A small amount of random noise $E_{simu}$, which satisfied the Gaussian distribution, was added to the simulated $Y_{simu}$ in order to obtain more realistic values $Y_{noise}$.

(17) $$Y_{noise}=AX_{simu}+E_{simu}$$

To facilitate the comparison of the ASCMART method with the SCMART and MART methods, the initial values are needed to operate the three algorithms. In this work, a different model such as NeQuick was selected as the background.

In order to evaluate the accuracy of the tomographic results of the three algorithms, the average density (AVD) error and root mean square (RMS) error were used in this work. They can be written as

(18) $$AVD=\frac{\sum_{d=1}^{n}\left|x_{d}^{IRI}-x_{d}^{ASCMART}\right|}{n}$$

(19) $$RMS=\sqrt{\frac{1}{n}\overset{n}{\underset{d=1}{\sum }}{\left(x_{d}^{IRI}-x_{d}^{ASCMART}\right)}^{2}}$$

**Figure 3:**
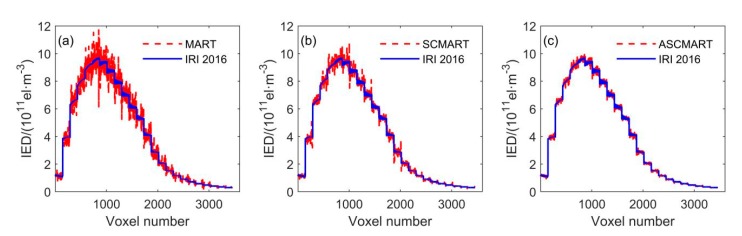
Comparison between the simulated ionospheric electron density distribution of the IRI 2016 model and the tomographic results of the three algorithms. (**a**) MART (**b**) SCMART (**c**) ASCMART.

Using the above discretized scenario, the reconstructed space was divided into 3456 voxels. In the numerical simulation, the IRI 2016 model was applied to simulate the true value of all voxels. At the same time, the initial value of each voxel was obtained from NeQuick model in order to perform the algorithms and distinguish the true value. Figure 3 compares the simulated result of the IRI 2016 model with the tomographic results of the MART, SCMART and ASCMART methods. From Figure 3c, we can see that the tomographic result of the ASCMART algorithm is in good agreement with the true value. This suggests that the ASCMART can be used to reconstruct the IED distribution. Comparing Figure 3c with Figure 3a,b, it can be seen that the result of the ASCMART is more consistent with the true value than those of the SCMART and the MART. 

Table 1 provides the reconstructed error statistics of the three algorithms based on all voxels. The RMS error of the ASCMART is 7 × 10^9^ el/m^3^, which is smaller than those of the MART and SCMART. 

**Figure 4:**
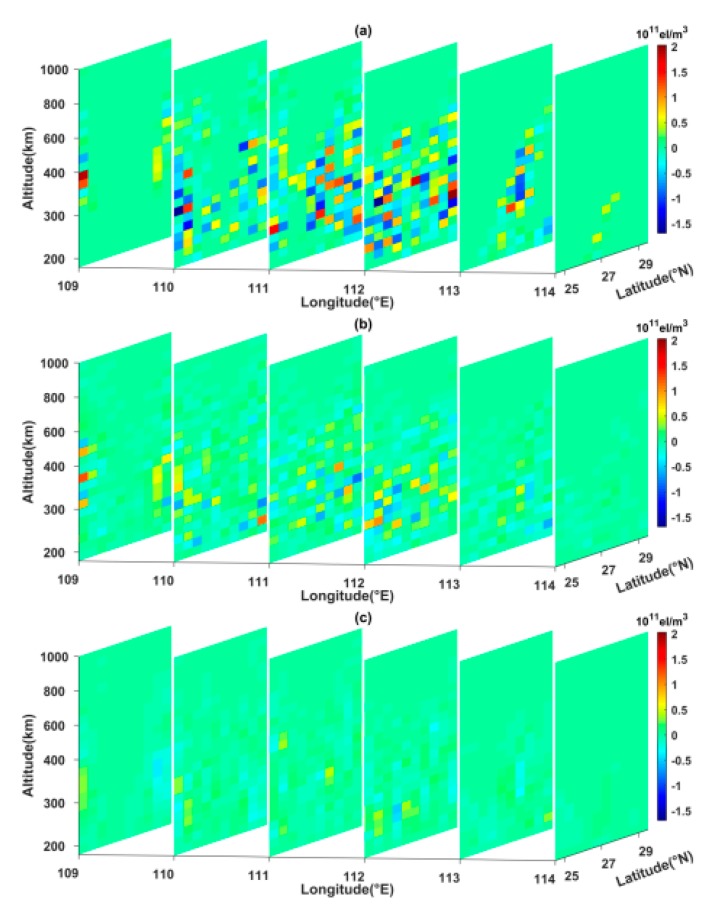
Reconstructed error comparison of the three algorithms along different longitudinal chains. (**a**) MART; (**b**) SCMART; (**c**) ASCMART.

Figure 4 illustrates the reconstructed error contour of the three algorithms along different longitudinal chains. From Figure 4, it can be seen that the reconstructed error of the ASCMART is the smallest of the three algorithms. Figure 4c and Table 1 show that the tomographic accuracy of the ASCMART method is the highest of the three methods, which confirms the reliability and superiority of the ASCMART.

To evaluate the reconstructed efficiency of the three algorithms, the iteration convergence condition was defined as follows in this work:

(20) $$S=\frac{\|x_{true}-x_{tomo}\|{}_{2}}{\|x_{tomo}\|{}_{2}}$$

where $x_{true}$ and $x_{tomo}$ represent the true value vector and the final tomographic solution vector of IED distributions, respectively.

**Figure 5:**
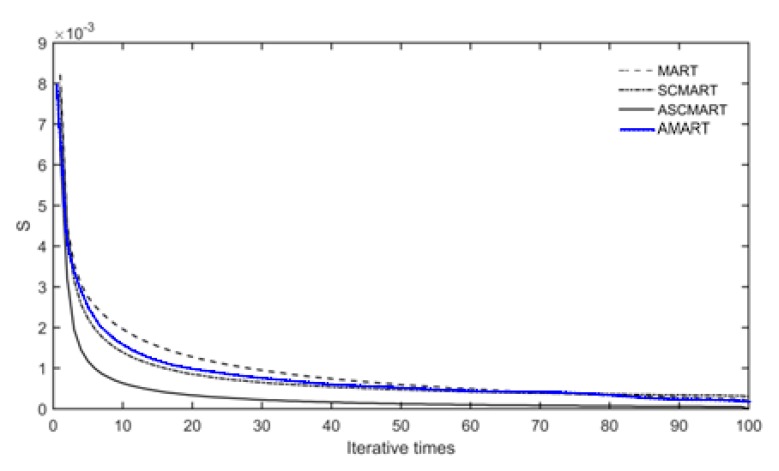
Comparison of the convergence speed of ASMART, SCMART, AMART, and MART.

In the simulation, the convergence of the iteration was terminated when S < 10^−3^. Since the AMART improved the convergence speed of the MART, the AMART was added to compare with the three algorithms. Figure 5 illustrates the convergence curves of the four algorithms. From Figure 5, we can see that the convergence speed of the ASCMART algorithm is faster than those of the SCMART, AMART, and MART. This means that the computational efficiency of the ASMART is the fastest in the four algorithms.

## 5. Tomographic Reconstruction of IED Distribution Using Actual GNSS Data

**Figure 6:**
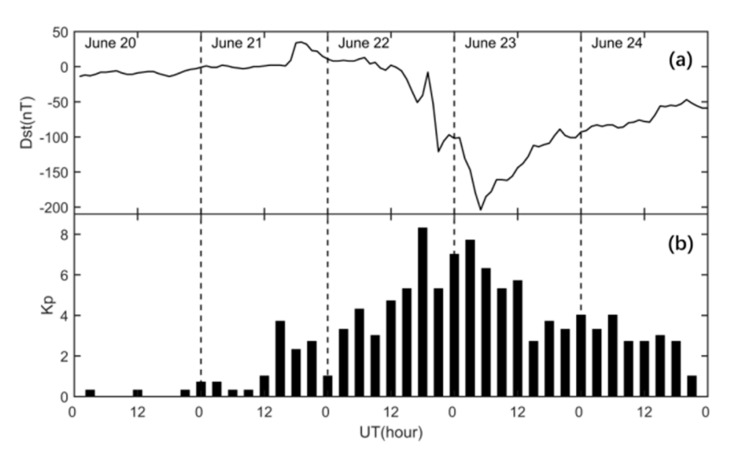
The variations in Dst index and Kp index from 20 to 24 June 2015.

Figure 6 shows the variations in the Dst and Kp indexes from 20 to 24 June 2015. There are three Kp indexes equaling to zero on 20 June 2015. From Figure 6, it can be seen that the Kp indexes are very small, and the Dst indexes remain near 0 on 20 June 2015. This means that the ionosphere activity is very quiet. At 15:00 UT on 22 June 2015, the Kp index reached 5+, and the Dst index began to decline. At 4:00 UT on 23 June, the Dst index reached the minimum value of −204 nt, and the Kp index again reached 6. From 15:00 UT on 22 June to 12:00 UT on 23 June 2015, the Kp indexes changed between 5 and 8. This indicates that a strong magnetic storm happened in this time period. 

To test the characteristics of the ASCMART by using actual observations, the observations of 124 CORS stations in Hunan province were introduced in this work, and the sample interval was 30 s. The selected time period was 20 June and 22–23 June 2015. Using the STEC derived from the selected dual-frequency GNSS data, the ASCMART method was applied to reconstruct the IED distributions in Hunan province. The IED temporal-spatial distributions under the two different ionospheric time periods were studied.

The STEC is the input data of the IED tomographic distributions. The reconstructed accuracy of IED depends on the STEC accuracy. Using the differential phases ($STEC_{\Phi }$) and the differential pseudoranges ($STEC_{p}$), an absolute STEC with high accuracy may be obtained by introducing an extra term $B_{L}$. It can be formulated as follows [32]:

(21) $$STEC=STEC_{\Phi }+B_{L}$$

If N measurements are obtained during a satellite pass, B_L_ can be modeled using the following equation:

(22) $$B_{L}=\sqrt{\overset{m}{\underset{c=1}{\sum }}{\left(STEC_{P_{c}}-STEC_{\Phi_{c}}\right)}^{2}/N}$$

To obtain high-accuracy STEC, the preprocessing of the GNSS data is very important. The method presented by Blewitt [33] was employed to clean outliers and repair cycle slips in GNSS observations. The differential phases was formed to eliminate the clock bias and the tropospheric error. In general, the instrumental biases of satellites and receivers were usually fixed in a single day. In this work, the instrumental bias was fitted using the least square technique.

**Figure 7:**
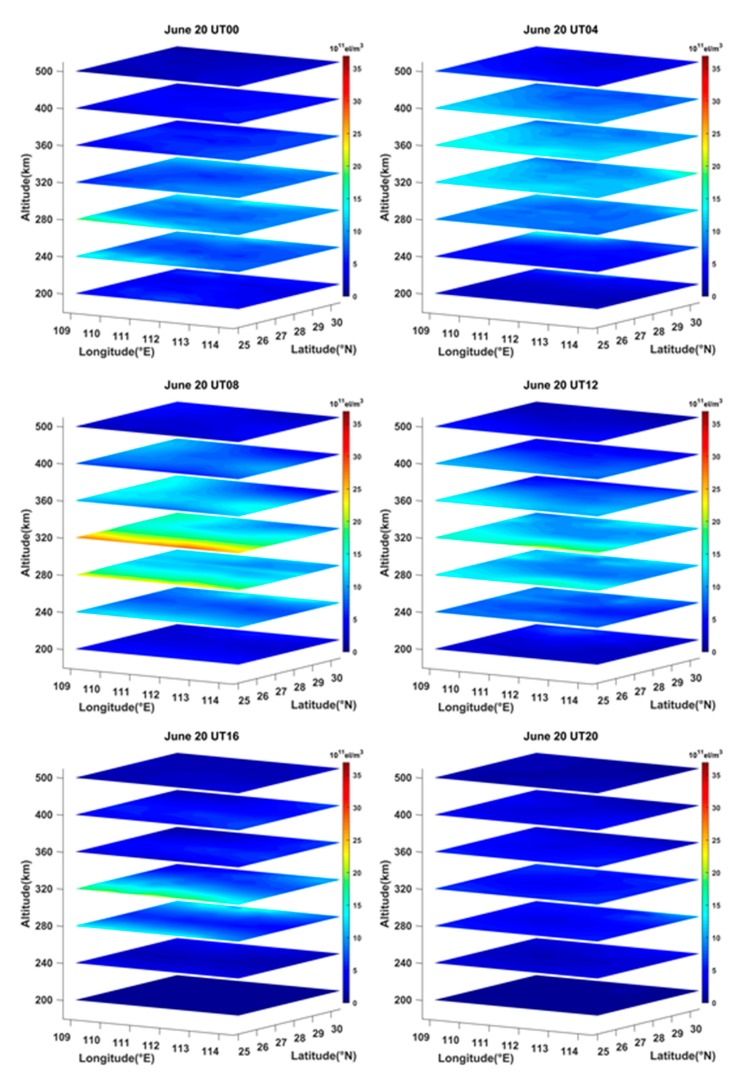
Time-series variation of three-dimensional IED distributions in the reconstructed region on 20 June 2015.

Using the STEC data derived from the selected GNSS observations, the ASCMART method was applied to reconstruct the three-dimensional IED distributions on geomagnetic quiet and geomagnetic storm days. The geomagnetic activity was very quiet on 20 June 2015. Figure 7 illustrates the three-dimensional IED distributions on the day. From Figure 7, it can be seen that the peak height of IED rises from 280 km to 360 km between 00:00 UT and 04:00 UT, and the peak height then falls to 320 km from 08:00 UT to 16:00 UT. At 20:00 UT, the peak height of IED falls to 280 km. The reconstructed images reflect the variations of the ionospheric vertical structure. Meanwhile, Figure 7 shows that the IED values first increase from 00:00 UT to 08:00 UT. As time goes on, the IED values gradually fall. At 20:00 UT, the IED values are the minimum. This is consistent with the normal change laws in daytime and nighttime over Hunan province, as well as the fact that the IED variation depends mainly on the intensity of solar radiation. In addition, we can see that the values of IED in the north of Hunan province are smaller than those in the south as a whole. This suggests that the IED distributions has strong correlation with latitude. 

**Figure 8:**
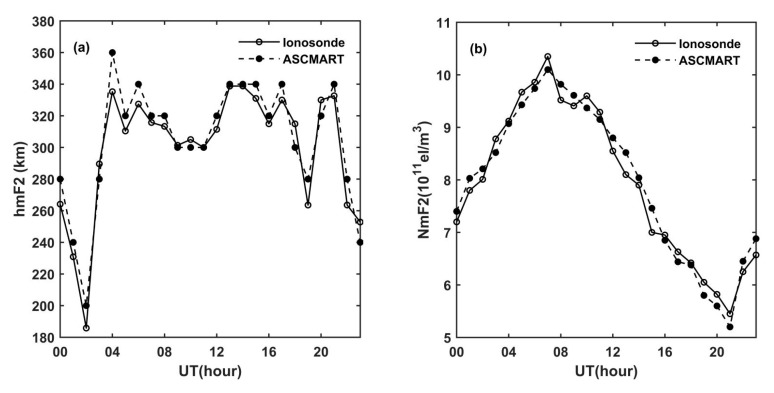
Comparisons of (**a**) the hmF2 and (**b**) NmF2 from the ASCMART with those obtained from the ionosonde located at Shaoyang on 20 June 2015.

To further verify the reliability of the tomographic results of the ASCMART, the ionospheric peak density hmF2 and the peak height NmF2 obtained from the ionosonde were compared with those of the ASCMART algorithm. In this work, we selected the ionosonde data from Shaoyang station, whose geographic location is shown in Figure 2. The comparison is shown in Figure 8. The comparisons confirm that the hmF2 and NmF2 extracted from the ASCMART algorithm have a good agreement with those obtained from the ionosonde as a whole. This suggests that the reconstructed results of the ASCMART are reliable.

To evaluate the superiority of the ASCMART, the IED reconstructed error can be obtained from the difference between ionosonde data and the results of the three algorithms at the geographic location of Shaoyang station. Table 2 shows the error statistics of the ASCMART, SCMART, and MART. From Table 2, it can be seen that the AVD error and the RMS of the ASCMART are the smallest among the three algorithms. This validates the ASCMART’s superiority compared to the other two algorithms.

**Figure 9:**
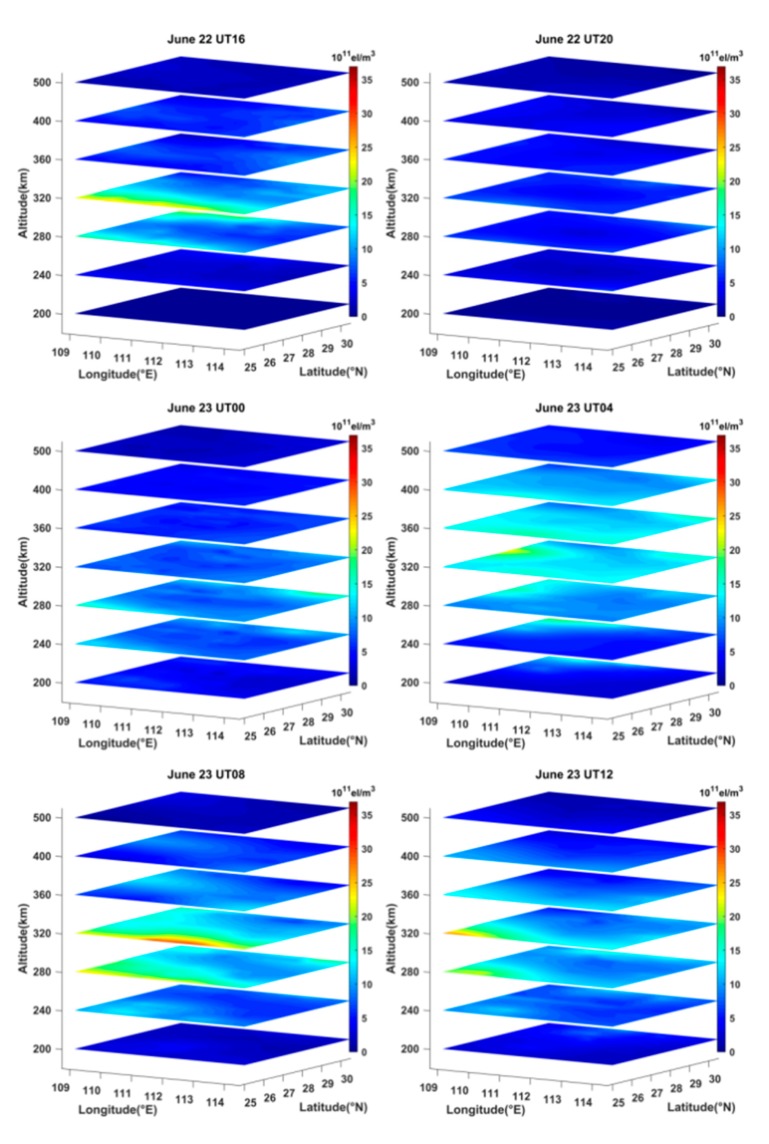
Time -series variation of three-dimensional IED distributions in the reconstructed region on 22 and 23 June 2015.

As mentioned above, a strong magnetic storm occurred between 15:00 UT on 22 June and 12:00 UT on 23 June 2015. We reconstructed the three-dimensional images of the IED distribution over Hunan province by using the ASCMART method and the GNSS data in this period. Figure 9 shows the time-series variation of the ionospheric structure on the selected geomagnetic storm days. 

**Figure 10:**
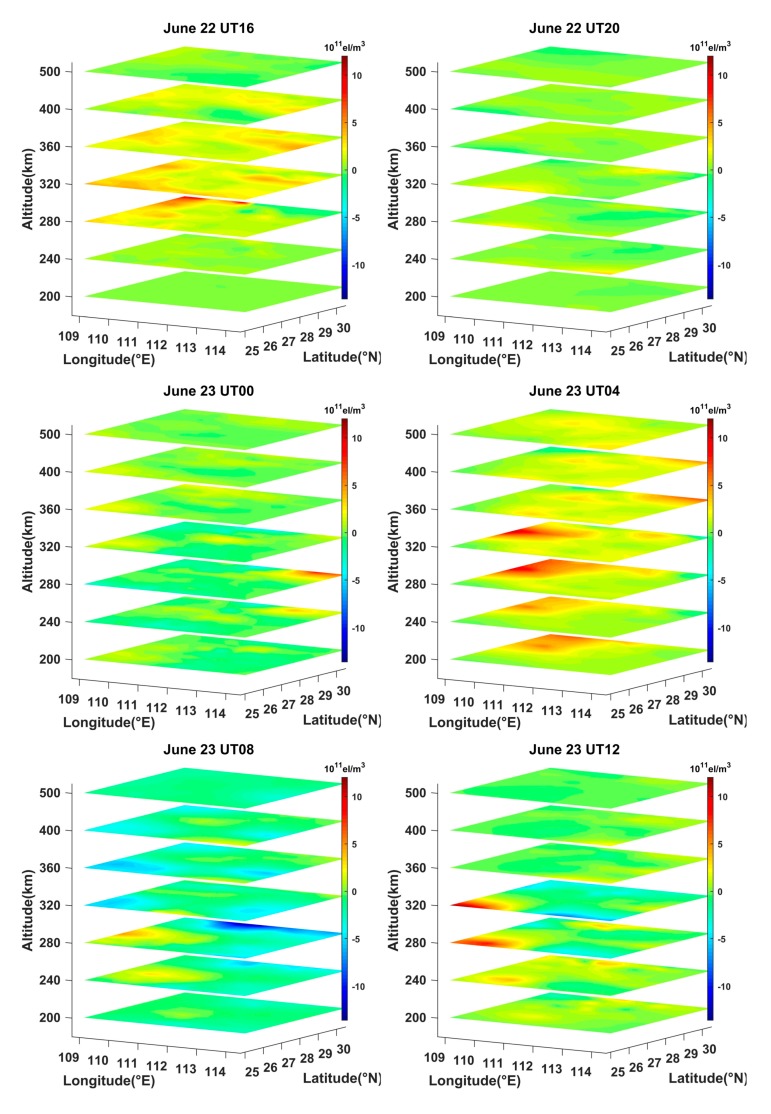
Time-series variation of three-dimensional differential IED in the reconstructed region on June 22 and 23, 2015.

Figure 10 illustrates the difference between the storm-time IED and the reference values, which are equal to the 15-day average before the storm occurrence. From Figure 10, it can be seen that the positive phase storm appeared between 260 km and 360 km at 16:00 UT on 22 June 2015. After that, the IED was almost unchanged between 20:00 UT on 22 June and 00:00 UT on 23 June. As time went on, the positive and the negative phase storm varied with time at different altitude. At 04:00 UT on 23 June, the strong positive phase storm appeared at altitudes of 200 km, 240 km, 280 km, and 320 km in the northwest of the reconstructed region, where the IED values increased obviously. At 08:00 UT on 23 June, the strong negative phase storm occurred at the altitude of 280 km in the north of Hunan province. The IED values increased slightly at the altitude of 240 km and 280 km but decreased at the altitude of 320 km and 360 km in the southwest of the reconstructed region. Afterwards, the IED showed almost no change except for a small-area increase at the altitude of 280 km and 320 km. This fact suggests that the reconstructed results of the ASCMART can reflect the storm phase variations with the altitude.

**Figure 11:**
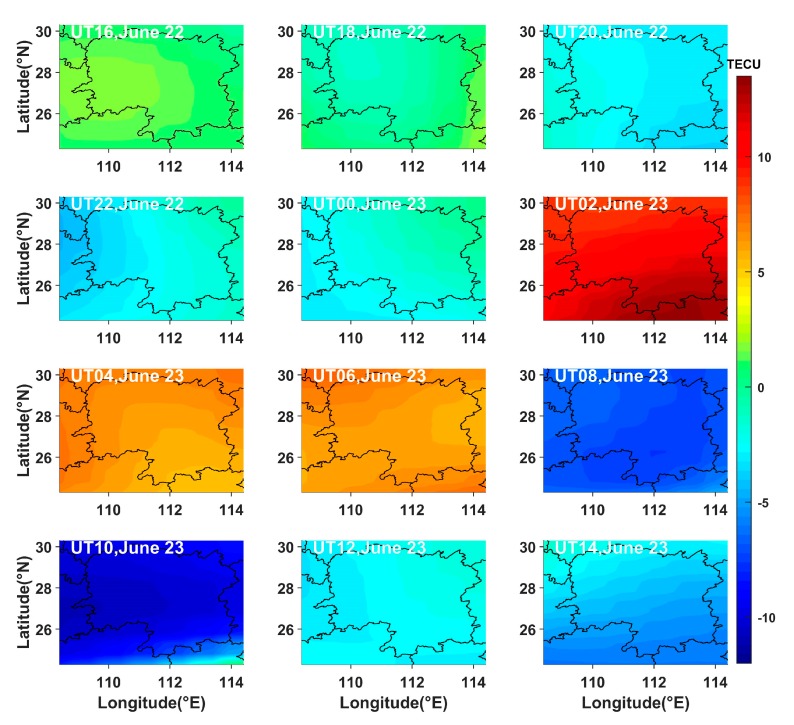
Time-series variation of differential total electron content on 22 and 23 June 2015.

To further characterize the temporal variations of the total electron content (TEC) maps during the storm, the TEC values were obtained by using the GNSS observations in Hunan province during the storm occurrence, and we calculated the differences between the above TEC values and the 15-day average TEC values before the storm occurrence. Figure 11 presents the time-series variations of the differential TEC values in the reconstructed region on 22 and 23 June 2015. Figure 11 indicates that the differential TEC values showed a small increase between 16:00 UT and 18:00 UT on 22 June. As time went on, the differential TEC values increased significantly from 02:00 UT to 06:00 UT on 23 June. However, the differential TEC values decreased greatly from 08:00 UT to 10:00 UT on 23 June and subsequently recovered to the normal state. The variation of the differential TEC values is generally in agreement with that of the IED change.

**Figure 12:**
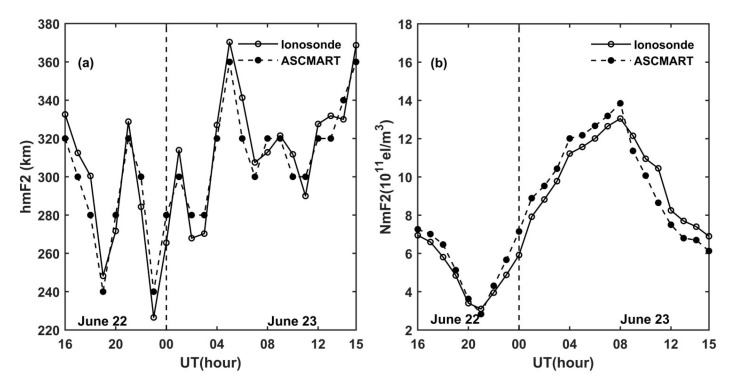
Comparisons of (**a**) the hmF2 and (**b**) NmF2 from the ASCMART with those obtained from the ionosonde located at Shaoyang on 22 and 23 June 2015.

To validate the reliability of the ASCMART during geomagnetic storms occurrence, the comparison of hmF2 and NmF2 was made between the ionosonde and the ASCMART. Figure 12 shows the comparison results; it can be seen that the hmF2 and NmF2 of the ASCMART have good agreement with those obtained from the ionosonde. This validates the feasibility and the reliability of the ASCMART to reconstruct three-dimensional images of the ionosphere under geomagnetic disturbance. 

Table 3 shows the error statistics of the four algorithms. Accordingly, we can see that the reconstructed accuracy of the ASCMART is higher than that of the SCMART and MART algorithms. This confirms that the ASCMART has superiority over the SCMART and MART.

## 6. Conclusions

In order to resolve the ill-posed problem of GNSS-based ionospheric tomography and the limitation of the conventional MART and the SCMART, a new ionospheric algorithm is presented in this work. The algorithm surmounts the dependence on the initial values for the voxels that have rays traversing them by imposing a horizontal and vertical smoothness constraint on the tomographic system, which improves the reconstructed accuracy of the IED distributions. Furthermore, the computational efficiency is improved by adaptively adjusting the constraint weight coefficients. The reliability of the GNSS-based tomographic algorithm was further tested by using GNSS data under geomagnetic quiet and geomagnetic disturbance conditions. The time-series variations of the three-dimensional ionospheric structure were studied, and the results were reasonable and consistent with the general behavior of the ionosphere. The positive and negative phase storm effects were found during geomagnetic storm occurrence. Comparison with profiles obtained from ionosonde data showed good agreement, and the validity of the tomography results were confirmed.

## Figures and Tables

**Table 1:** Tomographic error statistics of the MART, SCMART, and ASCMART.

Algorithm	MART	SCMART	ASCMART
Maximum error (10^11^ el/m^3^)	3.55	1.62	0.52
Average density error (10^10^ el/m^3^)	3.20	1.75	0.40
Root mean square error (10^10^ el/m^3^)	2.20	1.60	0.70

**Table 2:** Tomographic error statistics of the MART, the SCMART, and the ASCMART during geomagnetic quite day.

Algorithm	MART	SCMART	ASCMART
AVD error (10^10^ el/m^3^)	2.86	1.53	0.84
RMS error (10^10^ el/m^3^)	2.32	1.17	0.55

**Table 3:** Tomographic error statistics of the MART, SCMART, and ASCMART during geomagnetic storm days.

Algorithm	MART	SCMART	ASCMART
AVD error (10^10^ el/m^3^)	3.12	1.97	0.90
RMS error (10^10^ el/m^3^)	2.53	1.21	0.63
